# What We Do Not Know About the Costs of Immunization Programs in Low- and Middle-Income Countries

**DOI:** 10.1016/j.jval.2020.08.2097

**Published:** 2021-01

**Authors:** Allison Portnoy, Stephen C. Resch, Christian Suharlim, Logan Brenzel, Nicolas A. Menzies

**Affiliations:** 1 Center for Health Decision Science, Harvard T.H. Chan School of Public Health, Boston, MA, USA; 2 Management Sciences for Health, Boston, MA, USA; 3 Bill & Melinda Gates Foundation, Seattle, WA, USA; 4 Department of Global Health and Population, Harvard T.H. Chan School of Public Health, Boston, MA, USA

## Abstract

• For many countries, there are limited data on the costs of running immunization services, and even less on the costs of increasing immunization coverage.
• When considering different approaches for scaling up coverage, countries and funders need to understand the marginal change in coverage produced, costs of introduction, and how cost and coverage effects change depending on programmatic context.
• Costing studies would benefit from improved, systematic reporting and leveraging ongoing program evaluation efforts to collect costing data. Long-term investments in the health system may allow for routine data collection and improved efficiency for budgeting and planning.

## Introduction

Evidence on immunization economics is a critical input for country immunization programs, particularly in low- and middle-income countries (LMICs) with government- and donor-funded vaccination programs. This evidence allows programs to budget for current services, plan for new vaccine introduction, and evaluate the efficiency of service delivery strategies. Evidence on immunization costs is also useful for international funders when making resource allocation decisions.^1^, ^2^, ^3^, ^4^ A large number of studies have reported on the effectiveness and cost-effectiveness of childhood immunization for reducing the impact of vaccine-preventable diseases in LMICs.^5^, ^6^, ^7^ Nevertheless, these analyses often frame vaccine implementation strategies in general terms and make assumptions regarding costs and operational success. Bias or uncertainty in cost estimates can significantly influence the results of cost-effectiveness and budget impact analyses,^8^ and there is a substantial gap in the empirical evidence available on immunization economics. We discuss the nature of this evidence gap, and propose solutions for closing it.

## Closing the Gap: The Costs of Immunization Services

**Figure 1:**
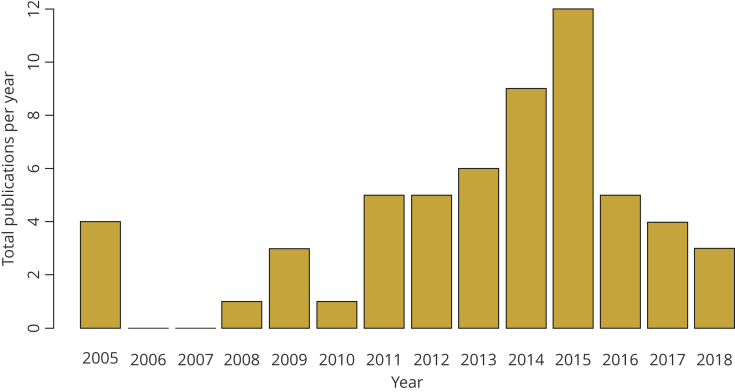
Annual number of unique publications reporting immunization costs, 2005-2018. Note: In total, the Immunization Delivery Cost Catalogue identified 61 unique publications reporting immunization unit costs for both routine services and supplementary immunization activities in the peer-reviewed and gray literature between 2005 and 2018, representing 33 different countries.^9^, ^10^

For most countries, available estimates of the costs of providing immunization services are uncertain, unreliable, old, or missing altogether. For example, the Immunization Costing Action Network’s recently developed Immunization Delivery Cost Catalogue identifies 61 unique publications reporting immunization unit costs for both routine services and supplementary immunization activities in the peer-reviewed and gray literature, representing only 33 different countries, and with only a small fraction of these data collected in the last 5 years (Figure 1).^9^^,^^10^ Although recent research investments have improved the availability of immunization costing data,^9^, ^10^, ^11^, ^12^ these efforts would still need to be greatly expanded to supply all countries with up-to-date and high-quality cost estimates.^13^ Because there is unlikely to be sufficient resources to conduct studies for all questions and settings of interest, researchers and decision makers need to weigh the resources required against both the magnitude of the evidence gap and the magnitude of the decisions being made. In practice, when immunization unit costs are needed for a new analysis, the necessary values are typically borrowed from neighboring countries, regional estimates, or global estimations to complete the analysis.^9^^,^^10^ Although this approach can be workable when there is a good understanding of the mechanisms that drive variation in cost estimates across settings,^4^ it can involve difficult subjective judgments when important contextual information is not reported, and strong assumptions about whether the approach used to impute the missing values is valid, often without accompanying sensitivity analysis to reflect the uncertainty in those assumptions. Although the low volume of costing research is a primary challenge, this gap has begun to close. Nevertheless, additional hurdles are introduced by the way studies are reported. Reporting of studies typically focuses on the specific research question, with less focus on the detailed reporting that would allow broader use of results. This issue of incomplete reporting in costing studies is commonly cited in the public health literature, and long recognized as affecting comparability of results across studies.^4^^,^^8^^,^^14^, ^15^, ^16^ Although some studies have developed and reported their results using systematic costing guidance,^11^ future studies across the public health sector would benefit from standardized reporting to get the most out of data collection. Although recent reference cases provide concrete guidance for implementing and reporting costing studies,^4^^,^^17^ it is unclear whether reporting practices have widely changed.

## The Remaining Gap: The Costs of Increasing Immunization Coverage

Although there are limited data on the costs of running immunization services,^9^, ^10^, ^11^ even less are available on the economics of increasing immunization coverage. As needed efforts to scale up immunization coverage for global disease eradication, elimination, and control remain ongoing,^5^^,^^18^ there is an accompanying need to collect and analyze the costs of scaling up to ensure efficient resource allocation and program management to improve coverage.^19^ Two systematic reviews of the incremental cost of scaling up immunization coverage in LMICs published in 2004 found that there was very limited evidence on the cost-effectiveness of approaches for improving immunization coverage.^20^^,^^21^ Little has changed in the 16 years since these reviews, with a recent systematic review finding 13 publications on the incremental costs of scaling up immunization coverage over 2003 to 2018 and coming to the same conclusion: the evidence was too scarce to aggregate in a meaningful way.^22^ A separate meta-analysis of these studies was able to identify a statistical relationship between current coverage level and the marginal costs of further coverage improvements, but only when data from high-income settings was included, and without being able to distinguish the different approaches used to achieve coverage improvements.^23^

For the continued support of immunization programs in LMICs, it is important to identify approaches that can be taken to improve immunization coverage. In addition to an understanding of what these approaches are, LMICs and funders need to understand the marginal change in coverage they produce, the costs of introducing them, and how these cost and coverage effects change depending on the programmatic context. There are already many ongoing efforts to improve immunization coverage that are being evaluated for programmatic effectiveness,^24^, ^25^, ^26^, ^27^, ^28^, ^29^ but there has been a missed opportunity to also measure the costs of these efforts. In a recent systematic review, 34 of the 41 full texts reviewed identified efforts to improve routine childhood immunization coverage, whereas 27 collected and reported effectiveness data on those efforts, 14 collected and reported costs, and 11 studies reported costs that permitted the estimation of an incremental cost-effectiveness ratio, albeit with caveats.^22^ Although collecting cost data alongside interventions of uncertain effectiveness and scalability may not demonstrate good value for money, an early understanding of the costs of these efforts is also important to judge future promise of scaling up coverage.

## A Way Forward

One potential solution to close the evidence gap for immunization economics would be to measure both the costs and effects of interventions, that is, conduct costing of discrete interventions designed to have incremental improvement in coverage that might support a generalizable cost function. Although routine data collection would also increase the reported cost data available, this approach would likely require health systems infrastructure improvements to support the routine collection of program expenditures and outputs. Additionally, such changes may lead to increased costs of introducing vaccines in LMICs, and would require consideration of the return on investment, compared to investing in the immunization program itself. In contrast, leveraging existing opportunities to collect costs alongside ongoing efforts to improve immunization coverage does not require health systems infrastructure improvements. Despite the need for useful cost data for immunization and other public health interventions, this solution has not been widely implemented. Although the addition of a cost component to ongoing immunization scale-up efforts is not trivial, it could also be quite valuable, particularly in addressing the costs of more intensive efforts to find unvaccinated children.^5^^,^^30^ Future research investigating the effects of efforts to improve coverage should include a detailed description of the intervention, as well as the costs of those efforts and any contextual factors that might affect impact and costs. For example, describing whether the intervention focuses on demand generation, vaccine delivery, novel technologies, or health systems strengthening, as well as contextual factors such as rurality, health care access and infrastructure, and health-seeking behaviors/vaccine hesitancy.^22^ Ideally, these evaluations would describe a causal model relating the various mechanisms thought to limit current coverage and how these are affected by the intervention.^31^ This model could then be used to investigate how intervention effects might generalize across settings. Such detailed reporting is required for extrapolating study evidence to other settings so that subsequent users of research findings can evaluate the relevant mechanisms of scale-up efforts rather than aspects local to a particular setting. Country-level decision makers considering activities to scale up immunization coverage using demand generation, for example, would then be able to refer to the cost-effectiveness of specific informational campaigns, such as home-based promotional education,^32^ community meetings and discussion groups,^33^^,^^34^ or targeted messaging of mothers with unvaccinated children.^35^ In the short term, costing studies will benefit from improved, systematic reporting and leveraging ongoing program evaluation efforts to collect costing data, while long-term investments in the health system and infrastructure may allow for routine data collection and improved efficiency for budgeting and planning.
